# Carbohydrate antigen 125 predicts pulmonary congestion in patients with ST-segment elevation myocardial infarction

**DOI:** 10.1590/1414-431X20199124

**Published:** 2019-12-05

**Authors:** F.J.A. Falcão, F.R.A. Oliveira, F. Cantarelli, R. Cantarelli, P. Brito-Júnior, H. Lemos, P. Silva, I. Camboim, M.C. Freire, O. Carvalho, D.C. Sobral-Filho

**Affiliations:** 1 Faculdade de Ciência, Educação e Tecnologia, Universidade de Pernambuco, Garanhuns, PE, Brasil; 2 Centro de Ciências Médicas, Hospital das Clínicas, Universidade Federal de Pernambuco, Recife, PE, Brasil; 3 Pronto Socorro Cardiológico de Pernambuco, Universidade de Pernambuco, Recife, PE, Brasil; 4 Unidade de Cardiologia Invasiva, Hospital Memorial São José, Rede D'Or São Luiz, Recife, PE, Brasil; 5 Instituto de Medicina Integral Prof. Fernando Figueira (IMIP), Recife, PE, Brasil

**Keywords:** Acute coronary syndrome, Pulmonary edema, CA125 antigen, Natriuretric peptide, Myocardial infarction

## Abstract

Carbohydrate antigen 125 (CA125) has long been used as an ovarian cancer biomarker. However, because it is not specific for ovarian cells, CA125 could also be used to monitor congestion and inflammation in heart disease. Acute heart failure (HF) is used to identify patients with a worse prognosis in ST-segment elevation myocardial infarction (STEMI). We aimed to determine the association of CA125 with acute HF in STEMI and to compare CA125 with N-terminal pro brain natriuretic peptide (NTproBNP) with a cross-sectional study. At admission, patients were examined to define Killip class and then underwent coronary angioplasty. Blood samples, preferably taken in the hemodynamic ward, were centrifuged (1500 *g* for 15 min at ambient temperature) and stored at −80°C until biomarker assays were performed. Patients were divided into two groups according to the presence or absence of congestion. Patients in Killip class ≥II were in the congestion group and those with Killip <II in the absence of congestion group. We evaluated 231 patients. The mean age was 63.3 years. HF at admission was identified in 17.7% of patients. CA125 and NTproBNP levels were higher in patients with Killip class ≥II than those with Killip class <II (8.03 *vs* 9.17, P=0.016 and 772.45 *vs* 1925, P=0.007, respectively). The area under the receiver operator characteristic curve was 0.60 (95%CI 0.53−0.66, P=0.024) for CA125 and 0.63 (95%CI 0.56−0.69, P=0.001) for NTproBNP. There was no statistical difference between the curves (P=0.69). CA125 has similar use to NTproBNP in identifying acute HF in patients presenting with STEMI.

## Introduction

Acute heart failure (HF) identifies patients with a worse prognosis in ST-segment elevation myocardial infarction (STEMI). The Killip classification, which is used to evaluate the severity of congestion through physical findings of ventricular dysfunction, predicts short- and long-term mortality. The higher the Killip class at presentation, the higher the risk of mortality (1–3).

However, congestion can be difficult to assess, especially when additional pulmonary signs are mild, such as acute HF due to STEMI. Physical assessment can detect only a moderate to high level of congestion. Signs and symptoms of congestion are caused by a series of events that begin with left ventricular dysfunction. Hence, hemodynamic congestion (elevation of cardiac pressures) precedes clinical congestion. The gold standard for evaluating hemodynamic congestion is the measurement of right atrial and pulmonary capillary wedge pressures (4). Unfortunately, no single noninvasive test can accurately detect hemodynamic congestion (5).

Carbohydrate antigen 125 (CA125) is a tumor marker of ovarian cancer. However, it is not specific for ovarian cells. CA125 is produced by volume overload (serosal mechanical stress) and inflammatory stimulus, and could therefore be a marker for both. Heart diseases are intrinsically related to congestion and inflammation. CA125 has therefore been extensively explored in the context of patients with heart disease, especially HF (6). Despite this, data on acute coronary syndrome are limited. From a sample of just 47 patients with acute coronary syndrome (27 with STEMI), De Gennaro et al. (7) found that CA125 could be used to identify those who developed pulmonary congestion.

This study aimed to evaluate the relationship between CA125 and acute HF in STEMI patients and to compare CA125 with N-terminal pro brain natriuretic peptide (NTproBNP), an established marker.

## Material and Methods

### Patients and study design

This was a cross-sectional study at a single center. The diagnosis of STEMI was made based on the third universal definition (8). At admission, patients underwent a physical examination to define Killip class and then underwent coronary angioplasty. A Killip class I is defined as the absence of signs of pulmonary congestion or systemic hypoperfusion; class II, as the presence of rales in the lower half of the lung or as the presence of gallop heart sounds; class III is defined as the presence of rales in the upper half of the lungs; and class IV is cardiogenic shock (significant hypotension: systolic blood pressure <90 mmHg or requiring inotropes). Patients were divided into two groups according to the presence or absence of congestion (acute HF). Patients in Killip ≥II were in the congestion group and those with Killip <II in the absence of congestion group.

Demographic and clinical data were prospectively entered into a database. One experienced interventional cardiologist, who was blinded to the patients' clinical information, analyzed all quantitative coronary angiography data. Thrombolysis in myocardial infarction (TIMI) flow was assessed before and after the angioplasty (9). Coronary artery disease was defined as single vessel or multivessel disease, according to the number of epicardial arteries with at least one lesion measuring >50% of the stenosis diameter. Procedural success was defined as achieving a minimum stenosis diameter reduction to less than 10% of the infarct-related artery, along with TIMI flow ≥2 without angiographic complication (10).

Coronary anatomy complexity was evaluated using the residual SYNTAX score (rSS), which involves calculation of the Syntax Score after angioplasty (11). This score evaluates stenosis that has reduced by more than 50% the luminal diameter of vessels measuring ≥1.5 mm in diameter. The number and extent of lesions, the tortuosity of the affected segments, the presence of thrombus or calcification, total occlusion, and involvement of bifurcation or trifurcation were evaluated. The lesions to be considered for analysis were identified and selected by consensus. Each selected lesion was then scored according to its complexity. The SS is the sum of the individual scores for each lesion and was calculated using the SYNTAX Score Calculator software version 2.11 (SYNTAX Score Working Group, http://www.syntaxscore.com).

The choice of access route and type of stent was left entirely to the discretion of the interventional cardiologist. Patients were excluded if they had chronic HF, previous coronary revascularization, ongoing infection, malignancy, end-stage liver disease, or kidney failure. The ethics committee of Instituto de Medicina Integral Prof. Fernando Figueira approved the study protocol. The study was performed in accordance with the ethical standards of the 1964 Declaration of Helsinki and its later amendments. Written informed consent was obtained from all individual participants included in the study.

### Laboratory measurements

Blood samples, preferably taken in the hemodynamic ward when the angioplasty access route was achieved, were placed in vacutainer test tubes according to the manufacturer's instructions (BD Vacutainer^®^, USA). The blood samples were then centrifuged (1500 *g* for 15 min at ambient temperature) and stored at -80°C until biomarker assays were performed. CA125 and NTproBNP were determined using commercially available kits (Roche, Germany). The manufacturer's cutoff values for pathological CA125 and NTproBNP test results are 35 U/ml and 125 pg/mL, respectively.

### Statistical analysis

Continuous variables are reported as means and standard deviations or as the median and the 25 and 75th percentiles, according to the presence or absence of a normal distribution, as evaluated using the Kolmogorov-Smirnov test. The Mann-Whitney test and Student's *t*-test were used for continuous variables in accordance with the distribution. Categorical variables are reported as absolute numbers and percentages. Fisher's exact test and Pearson's chi-squared test were used when appropriate.

The receiver operating characteristic (ROC) curve was used to determine the greatest area under the curve (AUC) in predicting pulmonary congestion of CA125 and NTproBNP. The AUC was compared with the DeLong test, using MedCalc Statistical Software, version 18 (MedCalc Software bvba, Belgium). All remaining statistical analyses were conducted using SPSS Statistics, version 21 (IBM, USA).

Univariate and multivariate regression analyses were used to identify potential independent predictors of congestion. Variables with a P value <0.2 in bivariate analysis were included in the model. The variables included in the model were age, gender, NTproBNP, CA125, systolic and diastolic blood pressure, heart rate, and rSS. The model was performed using backward stepwise selection. The model was accepted (P<0.001) and proved to be well adjusted according to the Lemeshow test (P=0.559) as it correctly classified 82.3% of cases. Odds ratios (OR) and their respective confidence intervals (95%CI) were used to quantify the effects. A final P value of less than 0.05 was considered significant.

## Results

We prospectively evaluated 231 patients admitted with STEMI undergoing coronary angioplasty between January 2018 and June 2018. The mean age in our sample was 63.3 years; 64.5% were men. Acute HF at admission was identified in 17.7% of patients. Table 1 shows the baseline characteristics of the study population according to pulmonary congestion. Age, arterial pressure, heart rate, initial TIMI flow, and rSS differed significantly between groups.

**Table 1:** Baseline clinical and angiographic characteristics of the entire study population according to pulmonary congestion.

Variables	Total study population (n=231)	Pulmonary congestion		P value
		No (n=190)	Yes (n=41)	
Age (years)	63.3±12.8	62.6±12.4	67.0±14.0	0.042
Male	149 (64.5%)	127 (66.8%)	22 (53.7%)	0.110
Hypertension	155 (67.1%)	126 (66.3%)	29 (70.7%)	0.585
Diabetes	66 (28.6%)	56 (29.5%)	10 (24.4%)	0.513
Dyslipidemia	69 (29.9%)	57 (30.0%)	12 (29.3%)	0.926
Current Smoker	102 (44.1%)	81 (42.6%)	21 (51.2%)	0.491
Systolic blood pressure (mmHg)	120 (100–145)	130 (105–150)	120 (80–140)	0.001
Diastolic blood pressure (mmHg)	80 (70–100)	80 (70–100)	75 (60–90)	0.014
Heart rate (bpm)	78 (64–97)	76 (64–88)	89 (70–104)	0.005
Ejection fraction	54.3±11.4	54.8±11.2	50.2±11.9	0.210
Anterior STEMI	108 (46.8%)	87 (45.8%)	21 (51.2%)	0.527
Initial TIMI flow				0.035
≤1	177 (76.6%)	140 (73.6%)	37 (90.2%)	
≥2	54 (23.4%)	50 (26.4%)	4 (9.8%)	
Angiographic success	164 (90.1%)	135 (90.6%)	29 (87.9%)	0.746
Multivessel disease	112 (60.9%)	88 (58.3%)	24 (72.7%)	0.123
rSS	8.4±8.2	7.8±7.3	11.3±9.6	0.045

Data are reported as n (%), means and standard deviations, and medians and 25 and 75th percentiles. Mann-Whitney test and Student's *t*-test were used for continuous variables. Categorical variables were compared with Fisher's exact test and Pearson's chi-squared test as appropriate. bpm: beats per minute; STEMI: ST elevation myocardial infarction; TIMI flow: thrombolysis in myocardial infarction flow; rSS: residual SYNTAX score.

**Figure 1:**
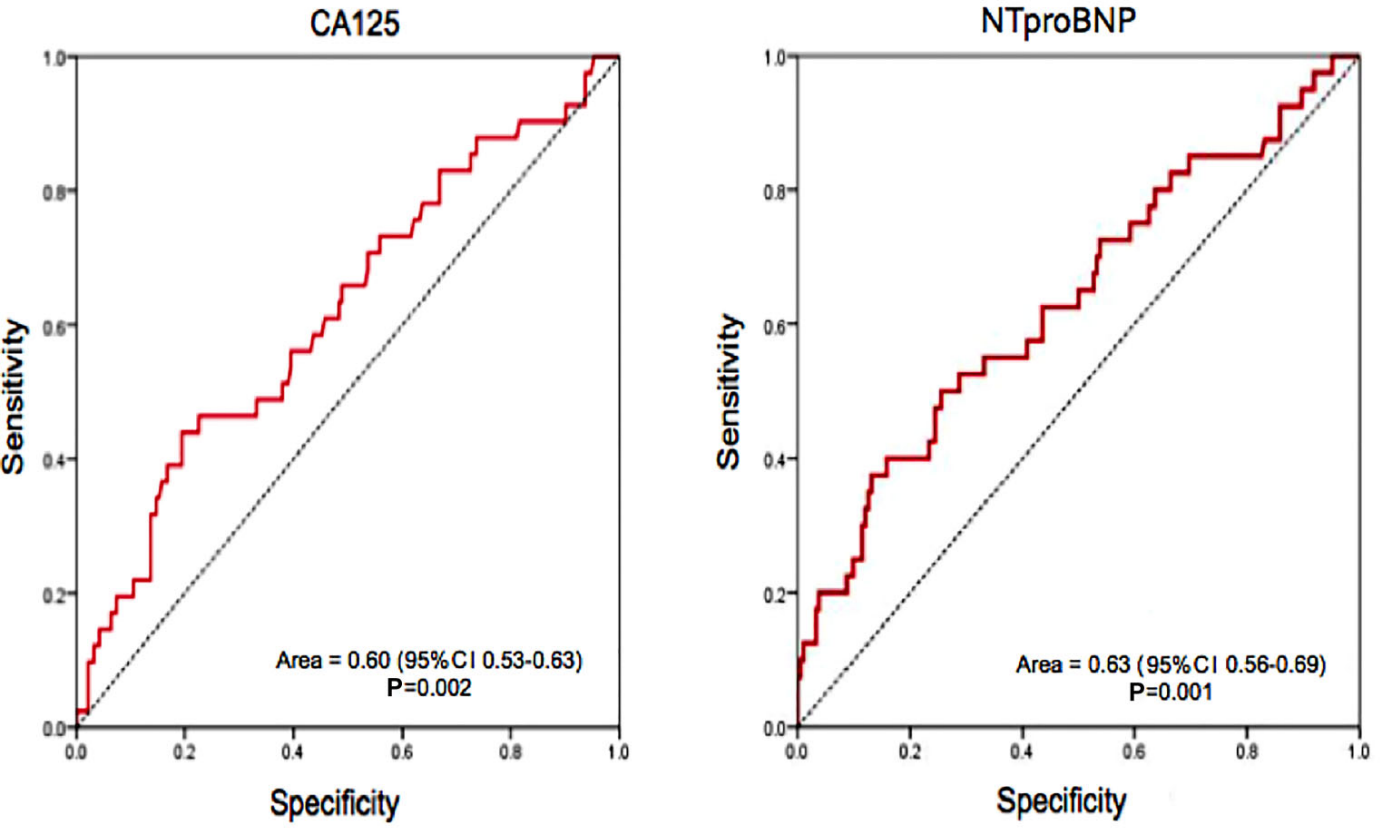
Receiver operating characteristic analysis of carbohydrate antigen 125 (CA125) and N-terminal pro brain natriuretic peptide (NTproBNP) for identifying pulmonary congestion.

CA125 and NTproBNP levels were higher in patients with pulmonary congestion (Table 2). The ROC curve analysis of CA125 and NTproBNP is shown in Figure 1. The optimal cutoff levels of CA125 and NTproBNP for predicting pulmonary congestion were 12.45 and 2010, respectively. The AUC was 0.60 (95%CI 0.53–0.66, P=0.002) for CA125 and 0.63 (95%CI 0.56–0.69, P=0.001) for NTproBNP. The comparison of ROC curves based on the difference between the AUCs demonstrated no statistical difference (difference between areas =0.027, P=0.69).

**Table 2:** CA125 and NTproBNP levels according to pulmonary congestion.

Biomarker	Pulmonary congestion		P
	No	Yes	
CA125 (U/mL)	8.03 (5.47–11.16)	9.17 (6.54–14.77)	0.016
NTproBNP (pg/mL)	772.45 (200.17–2096.50)	1925 (411.82–4688.25)	0.007

Data are reported as medians and 25 and 75th percentiles. Groups were compared with Mann-Whitney test. CA125: carbohydrate antigen 125; NTproBNP: N-terminal pro brain natriuretic peptide.

CA125 and NTproBNP levels at admission can be used to identify patients with pulmonary congestion with a sensitivity of 44 and 50% and specificity of 80 and 74.5%, respectively (Table 3).

**Table 3:** Performance of CA125 and NTproBNP in predicting pulmonary congestion.

	Optimal cutoff	Sensitivity (%)	Specificity (%)	PPV (%)	NPV (%)
CA125 (U/mL)	12.45	44	80	31	86
NTproBNP (pg/mL)	2010	50	74.5	28	87

CA125: carbohydrate antigen 125; NTproBNP: N-terminal pro brain natriuretic peptide; PPV: predictive positive value; NPV: negative predictive value.

Systolic blood pressure and heart rate were the only independent predictors of pulmonary congestion, based on multivariate regression analysis (Table 4).

**Table 4:** Independent predictors of pulmonary congestion according to multivariate regression analysis.

Variable	Univariate OR and 95% CI	P	MultivariateOR and 95% CI	P
Gender		0.157		0.168
Male	1.00		1.00	
Female	1.65 (0.83–3.30)		1.91 (076–4.80)	
NTproBNP (pg/mL)		0.011		0.118
≥2010	2.59 (1.25–5.36)		2.09 (0.83–5.59)	
<2010	1.00		1.00	
Systolic blood pressure	0.98 (0.97–1.00)	0.008	0.99 (0.97–1.00)	0.047
Heart rate	1.03 (1.01–1.05)	0.002	1.03 (1.01–1.06)	0.003
rSS	1.05 (1.00–1.10)	0.035	1.04 (0.99–1.10)	0.102

NTproBNP: N-terminal pro brain natriuretic peptide; rSS: residual SYNTAX score.

## Discussion

The major new finding in this study was that CA125 presented behavior similar to NTproBNP in patients with STEMI.

In 1967, Killip and Kimball published an article that stratified patients with STEMI based on physical examination. This classification is used to assess the severity of acute HF complicating myocardial infarction and predicts both short- and long-term mortality. The probable mechanism by which the Killip classification predicts events is the amount of myocardium in risk that is necessary to develop acute HF (1,12).

However, some physical signs of congestion are difficult to identify, especially in a critically ill patient. Auscultation of a galloping heart sound is relatively specific (90–97%) for elevated left ventricular end-diastolic pressure, but its sensitivity is low (9–51%) (13).

Killip's classification is a simple tool for risk stratification. However, biomarkers can be used to add significant prognostic information (14). Natriuretic peptides (NPs) are neurohormones specifically secreted from the cardiac chambers in response to volume and pressure overload that lead to increased wall tension. NP levels correlate with left ventricular dilatation, remodeling and dysfunction, congestive HF, and death among patients presenting with acute myocardial infarction (13,15–19).

CA125 is a glycoprotein belonging to the mucin family and is known as mucin (16 [Bibr B17] [Bibr B18]). Mucins are high molecular weight glycoproteins that protect epithelial surfaces through lubrication and hydration (20). Its half-life is approximately 7 days. It is a tumor marker for screening, diagnosing, and monitoring ovarian malignancy. Mechanical stress and inflammatory stimulus may release it from mesothelial cells. CA125 could therefore be a marker for congestion and inflammation in heart diseases.

Nägele et al. first described the relationship between tumor markers and clinical and hemodynamic findings in patients with chronic HF before and after cardiac transplantation. CA125 significantly correlated with filling pressures (right atrium and pulmonary capillary pressures) and with noradrenaline. There was a decrease in CA125 levels after transplantation or clinical stabilization and an increase when the patient's condition worsened (21). CA125 predicts chronic HF severity. Patients with New York Heart Association class I/II had lower CA125 levels than those with class IV (15±9 *vs* 167±94; P<0.005) (22).

CA1215 is a promising tool for risk stratification in heart disease, but there is a lack of evidence in STEMI patients. De Gennaro et al. (7) showed in 47 patients with ACS that CA125 at hospital admission could be used to identify those who would develop pulmonary congestion with a sensitivity of 42.7%, specificity of 97.1%, positive predictive value of 83.3%, and negative predictive value of 82.9%. Patients with pulmonary congestion were identified more accurately using CA125 than with BNP (83.3 *vs* 48.9%; P<0.001). The cutoff point of 25.3 U/mL was the best value to identify patients who presented pulmonary congestion on the ROC curve (sensitivity 58.3%, specificity 91.4%).

Rong et al. (23) studied 150 patients to evaluate the clinical value of CA125 in predicting short-term outcomes of coronary heart disease. However, the small number of deaths, only two, limited their ability to investigate the clinical prediction of this biomarker. The patients enrolled were likely at low risk for events.

To the best of our knowledge, this is the first study with only STEMI patients to demonstrate that CA125 is associated with congestion in a similar way to NTproBNP.

In this study, the diagnosis of pulmonary congestion was based on clinical signs (the Killip classification). Therefore, patients with only hemodynamic congestion could be considered in the group without pulmonary edema. De Gennaro et al. (7) used a chest X-ray to find a rate of 26% of pulmonary edema, higher than the rate we found. Signs of pulmonary congestion at chest radiography may even precede clinical symptoms. The relationship between the clinical diagnosis of pulmonary congestion and radiological findings has good specificity (78–99%), but low sensitivity (6–74%) (24). Furthermore, 44% of their patients did not have the diagnosis of STEMI, which makes comparison difficult.

The optimal CA125 cutoff level for predicting pulmonary congestion was 12.45, which differed greatly from the manufacturer's cutoff (<35 U/mL). This interval is based on the 99th percentile for a population of premenopausal women aged 18–40 years old and is used as an ovarian tumor marker. Levels related to events therefore vary depending on the clinical situation (6).

A congestion biomarker may objectively identify STEMI patients with ventricular dysfunction. Patients with pulmonary congestion are at higher risk of adverse outcomes and should be treated aggressively. NPs are the most widely used congestion marker. CA125 seems to be an alternative biomarker to substitute NPs in STEMI. Its wide availability and the fact that it costs approximately ten times less than NPs make it an emerging tool for stratifying heart patients. CA125 is not a direct marker of cardiac damage like troponin, nor a neurohormonal marker like NPs, but it is a marker of final organ damage caused by HF. Its relationship with congestion makes it a reliable marker of severity and therefore also of prognosis.

Some limitations must be considered. This was a single center study. The gold standard for evaluating congestion is the measurement of right atrial and pulmonary capillary wedge pressures. However, we did not compare these biomarkers of congestion with the gold standard. This is a surrogate endpoint study. We did not evaluate clinical endpoints such as re-hospitalization or mortality. These biomarkers were only measured at one time and the level might depend on the time from onset of chest pain.

In conclusion, CA125 had similar utility to NTproBNP for identifying acute HF in patients presenting with STEMI.
